# Patient-initiated versus fixed-interval patient-reported outcome-based follow-up in outpatients with epilepsy: a pragmatic randomized controlled trial

**DOI:** 10.1186/s41687-019-0151-0

**Published:** 2019-09-13

**Authors:** Liv Marit Valen Schougaard, Caroline Trillingsgaard Mejdahl, Jakob Christensen, Kirsten Lomborg, Helle Terkildsen Maindal, Annette de Thurah, Niels Henrik Hjollund

**Affiliations:** 1 AmbuFlex/WestChronic, Occupational Medicine, University Research Clinic, Aarhus University, Gl. Landevej 61, DK-7400 Herning, Denmark; 2 Department of Neurology, Aarhus University Hospital, Palle Juul-Jensens Boulevard 165, DK-8200 Aarhus N, Denmark; 3 Department of Clinical Medicine, Aarhus University, Palle Juul-Jensens Boulevard 82, DK-8200 Aarhus N, Denmark; 4 Department of Public Health, Aarhus University, Bartholins Allé 2, DK-8000 Aarhus, Denmark; 5 Department of Rheumatology, Aarhus University Hospital, Palle Juul-Jensens Boulevard 59, DK-8200 Aarhus N, Denmark; 6 Department of Clinical Epidemiology, Aarhus University Hospital, Olof Palmes Allé 43-45, DK-8200 Aarhus N, Denmark

**Keywords:** Patient reported outcome measures, Randomized controlled trial, Ambulatory care, Outpatient clinics, hospital, Epilepsy

## Abstract

**Background:**

The use of patient-reported outcome (PRO) could potentially contribute to the reorganization of the health care system. AmbuFlex is a PRO system used in remote patient monitoring, in which questionnaires are sent to patients at fixed intervals. The PRO data are used by clinicians to decide whether patients need clinical attention. Better self-management and cost-saving follow-up activities may be achieved by letting patients initiate need of contact. We evaluated the effects of patient-initiated PRO-based outpatient follow-up on health care resource utilization, quality of care, and the patient perspective.

**Methods:**

We conducted a parallel two-arm pragmatic randomized controlled trial at the Department of Neurology, Aarhus University Hospital, Denmark. Outpatients with epilepsy (≥ 15 years old), attending fixed-interval PRO-based follow-up with web-based questionnaires, were randomly assigned in a ratio of 0.55:0.45 to either 1) patient-initiated PRO-based follow-up (open access telePRO) or 2) fixed-interval PRO-based follow-up (standard telePRO). The primary outcome was the number of outpatient hospital contacts related to epilepsy retrieved from a regional registry. Hospitals admissions and emergency room visits were also assessed. Secondary self-reported outcomes including general health, well-being, health literacy, self-efficacy, number of seizures, side effects, confidence, safety, and satisfaction were retrieved from questionnaires. Data were analyzed by the intention-to-treat and per-protocol approaches.

**Results:**

Between January 2016 and July 2016, 593 patients were randomized to either open access telePRO (*n* = 346) or standard telePRO (*n* = 247). At 18 months, no statistically significant differences were found between the arms regarding number of telephone consultations or outpatient visits. Patients in the open access arm had a slightly lower, statistically significant number of emergency room visits than patients in the standard arm. Self-reported mental well-being in the open access arm was slightly, statistically significantly lower than in the standard arm. Other secondary outcomes did not differ statistically significantly between arms.

**Conclusion:**

This study did not find, as hypothesized, less use of health care resources or improved patient self-management or satisfaction in the patient-initiated PRO-based initiative compared to fixed-interval PRO-based follow-up. Patient-initiated PRO-based follow-up may be used as an alternative to fixed-interval PRO-based follow-up in patients who prefer this approach, but there is insufficient evidence for recommending a system-wide shift to patient-initiated PRO-based follow-up.

**Trial registration:**

Registered 4 February 2016 with ClinicalTrials.gov: NCT02673580.

**Electronic supplementary material:**

The online version of this article (10.1186/s41687-019-0151-0) contains supplementary material, which is available to authorized users.

## Introduction

Health care systems are experiencing an increased volume of patients with chronic conditions concurrent with increased focus on patient involvement and patient self-management [1, 2]. The use of patient-reported outcome (PRO) measures in clinical practice could potentially contribute to reorganization of the health care system and support patient involvement. PRO is a measurement directly reported by the patients based on their own perceived symptoms and health status [3]. Santana et al. describe a theoretical framework outlining the potential effects of using PRO measures in the care of chronically ill patients [2]. According to this framework, letting patients contribute with self-reported information about the impact of their disease and its treatment can contribute to better communication, engagement, self-management, and patient outcomes [2]. A number of reviews including randomized controlled studies have found that the use of PRO measures in a clinical setting improved patient-clinician communication, patient satisfaction, and detection of patients’ functional and mental health problems [4–6]. Furthermore, PRO has the potential to inform clinical decision-making and support self-management [7–9]. Findings related to clinical patient outcomes are less consistent [4, 6].

In Denmark, a generic configurable PRO solution, AmbuFlex, uses PRO measures as the very basis for outpatient follow-up in several chronic and malignant diseases [8, 10] including follow-up for outpatients with epilepsy. Epilepsy is a chronic condition characterized by recurrent seizures affecting functional, mental, and social aspect of life [11, 12]. Studies have reported that persons with seizures have increased risk of mood disorders, reduced quality of life, and significantly more social stigma than persons with no seizures [12, 13]. These findings support the need for differential and individualized follow-up in the care for patients with epilepsy. Several PRO measures have been developed for use in patients with epilepsy at the aggregated level [14]; however, the evidence regarding use of PRO measures on the individual level is weak [15, 16]. In 2012, an epilepsy version of AmbuFlex was developed in Central Denmark Region, here called standard telePRO [8]. In standard telePRO, the patients receive fixed-interval questionnaires at home instead of having pre-scheduled appointments at the outpatient clinic. Based on an automated algorithm, the patients’ PRO measures are used to decide the need for clinical attention, potentially leading to fewer visits and thus less treatment burden for well-treated patients. If the patient needs attention, the PRO measures are used to support patient-clinician communication.

Standard telePRO may not be an adequate solution if the patients have a variable need of clinical attention. A more patient-centered solution based on patient preferences to decide the timing of a clinical contact may be even more beneficial to enhance patient involvement and management of own care. Several reviews of randomized controlled trials have investigated the effect of patient-initiated interventions in which patients have direct access to the outpatient clinic if needed [17–19]. In studies of patients with rheumatoid arthritis, inflammatory bowel disease, and breast cancer, no differences were seen regarding clinical- or patient-reported health outcomes between patient-initiated intervention groups and clinician-initiated control groups. Furthermore, some studies found higher patient satisfaction and lower use of health care resources in the patient-initiated model [17–19]. We have not been able to find any studies that use a patient-initiated model in patients with epilepsy and no studies that use PRO as the main access point to flag the need for clinical attention.

In this study we evaluated the effects of patient-initiated outpatient follow-up in patients with epilepsy. The specific aims were to compare utilization of health care resources, quality of care, and the patient perspective in two outpatient follow-up activities: patient-initiated PRO-based follow-up (open access telePRO) versus fixed-interval PRO-based follow-up (standard telePRO).

We hypothesized that the number of contacts would be lower, quality of care at least as good, patient self-management better, and patient evaluation of health service improved among patients in the open access telePRO arm compared with those in the standard telePRO arm.

## Methods

### Study design

This study was a parallel two-arm pragmatic randomized controlled trial in which the participants were allocated to either patient-initiated PRO-based follow-up (open access telePRO) or fixed-interval PRO-based follow-up (standard telePRO). The study was carried out among epilepsy outpatients at the Department of Neurology, Aarhus University Hospital in Denmark. Standard telePRO has been used at the department since 2012. In January 2016, approximately 2500 epilepsy outpatients were attending standard telePRO follow-up. The study followed the Consolidated Standard of Reporting Trial (CONSORT) guideline for reporting parallel group randomized trials [20] and the CONSORT PRO extension [21] (Additional file 1). A study protocol has been published [22].

### Participants and settings

Participants were included between January 2016 and July 2016. From January 2016, all patients in standard telePRO follow-up at the Department of Neurology, Aarhus University Hospital, received a baseline research questionnaire combined with the fixed-interval epilepsy questionnaire from the outpatient clinic. Patients could choose to respond via a paper or web version of the questionnaires. Clinicians assessed the fixed-interval epilepsy questionnaire according to their normal routine, but were blinded to the research questionnaire. Approximately 14 days after the patients filled in the research questionnaire, eligible participants were randomized to either open access telePRO or standard telePRO. Patients were eligible if they were ≥ 15 years old, had an epilepsy diagnosis or suspicion of epilepsy, were attending standard telePRO follow-up, and had filled in the last questionnaire via the Internet. Patients were excluded if they were paper respondents or if they had stop attending standard telePRO follow-up before randomization.

The study coordinator enrolled participants approximately once a week during the inclusion period. After randomization, participants in the open access arm received detailed written information about the intervention via surface mail sent by the study coordinator. Participants were requested to contact the study coordinator if they did not want to participate in the open access telePRO intervention and preferred to continue with standard care (standard telePRO). Standard arm participants continued standard telePRO and there was no change in the follow-up. Blinding of the randomization allocation was not possible for either participants or clinicians. Follow-up assessments were conducted approximately 18 months after randomization [22]. The rationale for 18 months was based on the fact that more than in 80% of patients attending standard telePRO follow-up, questionnaires were sent at fixed 12-months intervals. This means that patients in standard telePRO follow-up may not have had contact with the outpatient clinic before 12 months had passed; thus, a follow-up period longer than 12 months was required.

### Pre-randomization

According to the inclusion criteria, patients were pre-randomized [23] in a ratio of 0.55:0.45 to either open access telePRO or standard telePRO. In a pre-randomization design, patients in the intervention arm are informed about the allocation following randomization, and disappointment about the allocation in the control arm can be prevented [23]. The skewed randomization allocation was applied because of an expected higher number of drop outs in the open access arm compared to the standard arm [22]. A higher dropout rate in the open access arm was expected, since participation was voluntary and the participants could at any time during the study decide to continue standard telePRO, if, for example, they did not want to initiate contact to the clinic by themselves, but rather receive questionnaires at fixed intervals. To account for this, we decided to randomize 10% more patients to the open access arm, as this would enhance the statistical power of the per-protocol analysis [24]. We used simple randomization due to an expected large study population and did not block randomization or other procedures to help achieve balance in the number or characteristics of the participants in the two arms. Computer-generated randomization was used. The computer code was developed and integrated into the WestChronic/ AmbuFlex system (Additional file 3, page 11) [10].

### Interventions

#### Standard arm – standard telePRO (usual care)

In standard telePRO, patients filled in fixed-interval disease-specific questionnaires every 3, 6, or 12 months, which were used as a partly automatic tool to support the decision regarding whether the patient needed clinical attention at the present time [8]. In the questionnaire, all patients could request a telephone consultation or an appointment in the outpatient clinic, regardless of their response to the other questions in the questionnaire. The questionnaire development is described elsewhere [25], and the questionnaire can be found in the Additional file 2.

The patient’s response to the questionnaires was given a green, yellow, or red color by using a pre-defined automated algorithm [8, 25]. Green indicated no need of clinical attention, red indicated need of attention, whereas yellow indicated that the patient might need attention. Green responses were handled automatically by the server software, and a new questionnaire was automatically scheduled to be sent to the patient at the pre-defined fixed interval, for example, after 12 months. All yellow and red responses were shown on an alert list, available to the clinicians, who accessed the list daily. A red response indicated need of clinical attention, and the clinician contacted the patient as quickly as possible. Patients were either contacted by telephone or they received a face-to-face appointment. For yellow responses, patients were only contacted if the clinicians judged that it was necessary. The patient’s questionnaire response was graphically presented to the clinicians, who accessed all the yellow and red responses through the Electronic Health Record system together with other relevant data from the record (laboratory tests, medication, etc.) [8, 22].

#### Intervention arm – open access telePRO

For patients randomized to open access telePRO, patient contact with the outpatient clinic was based on the patient’s preferences. Patients were asked to contact the outpatient clinic by themselves when they felt it necessary. Thus, at any time during the follow-up period, these patients could indicate a need for contact with the outpatient clinic by filling in the disease-specific questionnaire (Additional file 2). For this purpose, an open access website ‘My Epilepsy’ was developed. The website contains four core elements to allow patients to: 1) answer a questionnaire when they needed to get in contact with the clinic, 2) view their previously questionnaire responses, 3) view information about the questionnaire, and 4) view contact information (e.g. telephone number) to the outpatient clinic [22]. Full detail of the development and features of this website are available elsewhere [22]. Patients had access to the open access website via a secure login to the Danish ehealth Portal “Sundhed.dk”. In addition, the patients could also phone the outpatient clinic if needed. All questionnaire responses in the open access arm turned red (definite need of attention) on the alert list to the clinicians, since these patients were instructed to only fill in the questionnaire if they needed to talk to a clinician. The clinician checked the alert list daily and assessed the red open access responses as quickly as possible in the same web-system as in standard telePRO [8, 22]. The patients were contacted by telephone, and a face-to-face appointment was scheduled if necessary. If the patient did not fill in a questionnaire to the outpatient clinic within a priori defined time-span, the web system automatically sent a reminder to the patients with instructions to fill in the questionnaire. For example, a reminder was sent after 12 months if the patient prior to randomization was originally referred to a 6-month fixed questionnaire interval in standard telePRO. The clinicians also received information on the alert list about patients who did not respond to these reminders, and they were subsequently contacted by a clinician.

### Outcomes

#### Primary outcome

The primary outcome was the number of outpatient hospital contacts related to epilepsy from baseline to follow-up (timeframe 18 months). The number of contacts included all outpatient telephone consultations and outpatient visits (face-to-face consultations) with a nurse or a physician. Data regarding hospital admissions and emergency room visits were also assessed. The number of telephone consultations, outpatient visits, hospital admissions, and emergency room visits during the 18-month period were retrieved separately from a regional registry: the Business Intelligence Register in Central Denmark Region, which contains information about routinely collected activity measures from the Department of Neurology and Aarhus University Hospital.

#### Secondary outcomes

Secondary self-reported outcomes were retrieved from the baseline research questionnaire and a follow-up research questionnaire sent to patients before randomization and 18 months after randomization. Both questionnaires included information about number of seizures, side effects, well-being, general health, health literacy, self-efficacy, patient activation, confidence, safety, and satisfaction.

##### Clinical outcome measures

The number of seizures last year and the degree of side effects were extracted from two single items in the epilepsy questionnaire (Additional file 2). Test-retest reliability of the side effects item has been reported to be substantial [25], but validity has not yet been reported. The side effects item ranges from 1 (best) to 4. Mortality was recorded at the end of the follow-up period and retrieved from the Business Intelligence Register in Central Denmark Region.

##### Patient-centered outcome measures

Well-being was extracted from the WHO-5 Well-Being Index (WHO-5) [26, 27]. WHO-5 is a generic questionnaire, and the psychometric findings have been reported in other patient populations [27]. The WHO-5 includes five items which are used to calculate a score that ranges from 0 (worst) to 100. General health (GH) was extracted from one single item: “In general, would you say your health is: excellent, very good, good, fair, or poor” from the generic questionnaire: The Short Form Health Survey SF-36 [28, 29]. The GH item was scaled from 1 (best) to 5.

##### Patient self-management

Health literacy was extracted from the generic Health Literacy Questionnaire (HLQ), sub-scale 4: “Social support for health”, sub-scale 6: “Ability to actively engage with healthcare providers”, and sub-scale 9: “Understand health information well enough to know what to do” [30, 31]. HLQ sub-scale 4 is a 4-item scale that ranges from 1 (worst) to 4, whereas HLQ sub-scales 6 and 9 are 5-item scales ranging from 1 (worst) to 5. Self-efficacy was extracted from the generic 10-item General Self-Efficacy Scale (GSE) [32, 33]. The psychometric properties of GSE have been evaluated across many countries [32]. The GSE score ranges from 10 (worst) to 40. Patient activation was extracted from two single items modified from a generic questionnaire: the Patient Activation Measure (PAM) [34]. The two PAM items: “I am confident that I can tell when I need to get outpatient care” and “I am confident I can figure out solutions when new situations or problems arise with my health condition” range from 1 (worst) to 4.

##### Patient health service evaluation

Confidence, safety, and satisfaction were extracted from three single items, which were modified from a patient-reported experiences questionnaire developed by the Danish Cancer Society [35]. Psychometric properties have not been reported. Scores for the three items range from 1 (best) to 4.

##### Other measurements

All Danish Citizens have a 10-digit unique personal identification number assigned to all citizens at birth [36]. It encodes gender and date of birth, and was used to calculate age and gender at baseline. Other patient characteristics were extracted from the baseline research questionnaire including cohabitation status, education, and duration of epilepsy. The education variable was categorized into three levels: no or low (primary and lower secondary school), medium (upper secondary school and short cycle tertiary), and high (bachelor and master). Duration of epilepsy was divided into two groups with a cut-off point at 2 years duration, as this was considered an acceptable level of being experienced or not-experienced with the epilepsy diagnosis.

##### Process evaluation

Automated computer logs were used to track and evaluate use of the open access website “My Epilepsy” in the WestChronic/AmbuFlex-system [10]. Use was defined as number of questionnaires filled in by the patients. Number of reminders mailed to patients and the number of patients who responded to these reminders were also logged into the system.

### Sample size

Based on a two-sided statistical test, the study was designed to have a power of 90% (*P*-value 0.05) [22]. This was based on a study that reported that the mean number and standard deviations (SDs) of outpatient visits were 4.12 (SD = 3.41) in the open access arm and 4.64 (SD = 2.38) in the control arm [37]. We expected to detect a difference of at least one contact between the arms. This required a sample size of 386 participants. To account for attrition and loss to follow-up, the sample size was supplemented with 207 patients (132 in the open access arm and 75 in the control arm).

### Statistical methods

Data were analyzed based on the intention-to-treat (ITT) approach. Between-arm differences in the number of outpatient visits, telephone consultations, hospital admissions, and emergency room visits were analyzed by simple linear regression. Because the normality distributions were skewed, 95% confidence intervals were found by using the bootstrap method with 1000 replications [38, 39]. Between-arm differences in all secondary outcomes, apart from mortality, were analyzed by multiple linear regression by calculating differences at follow-up (18 months) adjusted for the baseline value.

Between arm differences were also analyzed on a per-protocol basis. The per-protocol analysis included only participants who completed the open access intervention. Patients were defined as ‘completers’ if they did not decline to participate in the intervention during the study period. Between-arm differences in the number of outpatient visits, telephone consultations, hospital admissions, and emergency room visits were analyzed by multiple linear regression adjusted for gender, age, education, cohabitation status, epilepsy duration, and seizures during last year. Confidence intervals were found by using the bootstrap method with 1000 replications [38, 39]. Between-arm differences at follow-up of secondary outcomes were analyzed by multiple linear regression adjusted for the baseline value, gender, age, education, cohabitation status, epilepsy duration, and seizures during last year.

Differences in baseline data between the arms were evaluated by chi-squared test for categorical variables and the Wilcoxon Mann–Whitney test or unpaired *t*-test for continuous variables. Normally distributed baseline data were presented with means and SDs, otherwise medians and interquartile ranges (IQR) were reported additionally. In PRO measures, information about item nonresponse was presented as numbers and percentages. Estimation of sum scores followed guidelines for handling items missing for each specific score. In the HLQ scores, the mean scores of the other items were used to estimate the score. If more than two items were missing, the score was not estimated. This instruction was obtained from a user package we received after signing a license agreement. We were not able to find a standardized guideline for the other scales (GSE and WHO-5) and thus, we decided not to calculate the score if items were missing.

To explore whether the results changed in sub-groups in the population, we performed supplemental explorative ITT analyses by stratifying on age, gender, and high/low health literacy (HLQ4: “social support for health”). The median values for age (median value = 45.7 years) and the HLQ4 scale (median value = 3.4) at baseline were used to define the threshold of the high (≥ 45.7 years and ≥ 3.4 HLQ4 score) and low groups (< 45.7 years and < 3.4 HLQ4 score). Furthermore, ITT-sensitivity analyses were used to establish the impact of missing self-reported data at follow-up. Sensitivity analyses were only performed for the WHO-5 score. If the WHO-5 score was missing at follow-up, the score was imputed by using the WHO-5 score from the baseline research questionnaire. Four scenarios of the imputed follow-up values were considered: 1. the baseline value was reduced with 5 points in the open access arm and was unchanged in the standard arm, 2. the baseline value was reduced with 5 points in the standard arm and was unchanged in the open access arm, 3. the baseline value was increased with 5 points in the open access arm and was unchanged in the standard arm, and 4. the baseline value was increased with 5 point in the standard arm and was unchanged in the open access arm. Then, between-arm ITT-differences in the WHO-5 score at follow-up were analyzed by multiple linear regression adjusted for the baseline WHO-5 value. All analyses were conducted in STATA version 15 (Stata Corporation, College Station, Texas, USA).

## Results

### Participant flow and baseline data

**Fig. 1:**
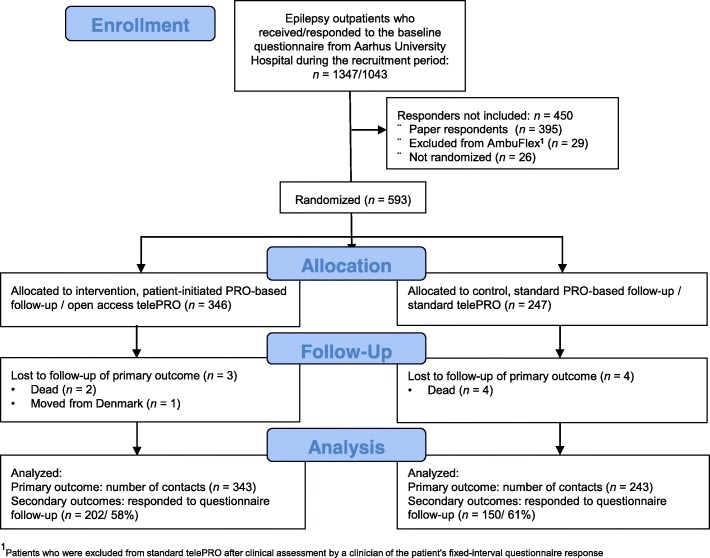
The study CONSORT flow diagram

A total of 593 outpatients with epilepsy were included from January 2016 to July 2016; 346 were randomized to the open access telePRO arm and 247 to the standard telePRO arm (Fig. 1). A total of six patients died (two patients in the open access arm and four patients in the standard arm), and one patient from the open access arm moved abroad within the follow-up period of 18 months: these seven patients were not included in the analyses. With respect to secondary self-reported outcomes, 202 (58%) in the open access arm and 150 (61%) in the standard arm responded to the questionnaire at both baseline and at follow-up. During the follow-up period, 43 patients declined to participate in the open access intervention and were excluded in the per-protocol analyses. The baseline characteristics of the participants are shown in Table 1. No statistically significant baseline differences were found between the open access arm and the standard arm. Also, no statistically significant differences were found between patients who completed the open access intervention (the per-protocol arm) and the standard arm.

**Table 1:** Participant baseline characteristics, *N* = 593

Variables	Intention-to-treat population		Per-protocol population
	Intervention arm (open access telePRO): *n* = 346	Control arm (standard telePRO): *n* = 247	Patients who completed the open access intervention: *n* = 300
Mean (SD) age, years	46.3 (17.2)	47.2 (17.3)	45.8 (17.1)
Gender: male*, n* (%)	182 (53)	115 (47)	164 (55)
Cohabitation status, *n* (%)			
Living alone	76 (22)	62 (25)	59 (20)
Missing	12 (3)	3 (1)	12 (4)
Education, *n* (%)			
No or low	94 (27)	62 (25)	76 (25)
Medium	119 (34)	96 (39)	105 (35)
High	121 (35)	85 (34)	107 (36)
Missing	12 (3)	4 (2)	12 (4)
Duration of epilepsy, years			
Mean (SD)	16.1 (14.3)	16.9 (15.7)	16.2 (14.4)
Median (IQR)	12 (5–22)	12 (5–22)	11 (5–23)
Missing, *n* (%)	46 (13)	29 (12)	41 (14)
Number of seizure last years, *n* (%)			
No seizure	235 (68)	165 (67)	213 (71)
Seizures (1 or above)	96 (28)	69 (28)	74 (25)
Missing	15 (4)	13 (5)	13 (4)
Side effects			
Mean (SD)	1.56 (0.79)	1.45 (0.67)	1.53 (0.76)
Median (IQR)	1 (1–2)	1 (1–2)	1 (1–2)
Missing, *n* (%)	7 (2)	2 (1)	7 (2)
Well-being (WHO-5)			
Mean (SD)	68.9 (18.9)	68.0 (19.4)	69.0 (18.9)
Median (IQR)	72 (60–80)	72 (56–80)	72 (60–80)
Missing, *n* (%)	10 (3)	3 (1)	9 (3)
General health			
Mean (SD)	2.62 (0.92)	2.64 (0.91)	2.59 (0.92)
Median (IQR)	3 (2–3)	3 (2–3)	3 (2–3)
Missing, *n* (%)	7 (2)	3 (1)	7 (2)
Social support for health (HLQ, subscale 4)			
Mean (SD)	3.30 (0.54)	3.32 (0.56)	3.30 (0.55)
Median (IQR)	3.4 (3–3.8)	3.4 (3–3.8)	3.4 (3–3.8)
Missing, *n* (%)	15 (4)	6 (2)	14 (5)
Ability to actively engage with healthcare providers (HLQ, subscale 6)			
Mean (SD)	3.87 (0.84)	3.82 (0.91)	3.87 (0.84)
Median (IQR)	4 (3.4–4.5)	4 (3.4–4.6)	4 (3.4–4.4)
Missing, *n* (%)	18 (5)	6 (2)	16 (5)
Understanding health information well enough to know what to do (HLQ, subscale 9)			
Mean (SD)	4.01 (0.79)	3.94 (0.86)	4.02 (0.77)
Median (IQR)	4 (3.6–4.6)	4 (3.6–4.6)	4 (3.6–4.6)
Missing, *n* (%)	18 (5)	6 (2)	16 (5)
Self-efficacy (GSE)			
Mean (SD)	29.35 (6.08)	29.23 (6.73)	29.71 (5.86)
Missing, *n* (%)	16 (5)	7 (3)	15 (5)

Differences between the arms were evaluated by chi-squared test, Wilcoxon Mann–Whitney test, or unpaired *t*-test. No *p*-value < 0.05 was found

*SD* Standard deviation, *IQR* Interquartile range, *WHO-5* WHO-5 well-being index, *HLQ* Health literacy questionnaire, *GSE* General self-efficacy scale

### Primary outcomes

No statistically significant differences were found between the arms regarding mean number of telephone consultations or outpatient visits (Table 2). The mean difference in telephone consultations between the open access arm and the standard arm was − 0.32 (95% CI: − 0.68 to 0.05). Patients in the open access arm had a statistically significant, slightly lower number of emergency room visits than those in the standard arm; the mean difference was − 0.11 (95% CI: − 0.21 to − 0.01). No statistically significant difference was found in hospital admissions.

**Table 2:** Healthcare utilization during an 18-month follow-up period among outpatients with epilepsy

Primary outcome	Intention-to-treat population			Per-protocol population	
	Intervention arm (open access telePRO): *N* = 343	Control arm (standard telePRO): *N* = 243	Mean difference (95% CI)	Completed the open access intervention: *N* = 300	Mean difference (95% CI)
Outpatient visits ^a^					
Mean (SD)	0.45 (0.95)	0.42 (0.86)	0.03 (−0.11 to 0.18)	0.43 (0.91)	0.04 (−0.12 to 0.20)
Median (Range)	0 (0–7)	0 (0–6)		0 (0–7)	
Telephone consultations ^a^					
Mean (SD)	0.99 (1.88)	1.30 (2.46)	−0.32 (− 0.68 to 0.05)	0.90 (1.80)	−0.20 (− 0.55 to 0.15)
Median (Range)	0 (0–12)	1 (0–22)		0 (0–12)	
Hospitalizations ^a^					
Mean (SD)	0.05 (0.29)	0.09 (0.49)	−0.04 (− 0.10 to 0.03)	0.05 (0.25)	0.0002 (−0.05 to 0.05)
Median (Range)	0 (0–3)	0 (0–5)		0 (0–2)	
Emergency room visits ^b^					
Mean (SD)	0.07 (0.38)	0.19 (0.72)	−0.11 (− 0.21 to −0.01)	0.06 (0.31)	−0.08 (− 0.18 to 0.007)
Median (Range)	0 (0–4)	0 (0–7)		0 (0–3)	

The estimated intention-to-treat mean differences and 95% CIs were obtained after simple linear regression by using the bootstrap method with 1000 replications [39]

The estimated per-protocol mean differences and 95% CIs were obtained after multiple linear regression adjusted for gender, age, education, cohabitation status, epilepsy duration, and seizures last year by using the bootstrap method with 1000 replications [39]

*SD* Standard deviation, *CI* Confidence interval

^a^at the Department of Neurology, Aarhus University Hospital, ^b^ at Aarhus University Hospital

### Secondary outcomes

No statistically significant differences were found between the open access arm and the standard arm regarding clinical outcome measures such as seizures during the last year, side effects (Table 3), and mortality. Patient-centered outcome measures showed a statistically significant difference of − 3.21 (95% CI: − 6.38 to − 0.05) in the WHO-5 well-being score at follow-up, giving a lower score in the open access arm than in the standard arm. General health status did not differ between the two arms. Furthermore, no statistically significant differences were found in outcome measures related to patient self-management (health literacy, self-efficacy, patient activation) and health service evaluation (confidence, safety, satisfaction).

**Table 3:** Patient-reported outcomes measured 18 months after randomization among outpatients with epilepsy

Secondary outcomes	Intention-to-treat population			Per-protocol population	
	Intervention arm (open access telePRO): *N* = 202	Control arm (standard telePRO): *N* = 150	Difference ^a^ at 18- mo. follow-up (95% CI)	Completed the open access intervention: *N* = 195	Difference ^b^ at 18- mo. follow-up (95% CI)
Well-being (WHO-5)					
Mean (SD)	66.99 (19.45)	69.29 (18.01)	−3.21 (−6.38 to −0.05)	66.94 (19.64)	−3.26 (−6.68 to 0.16)
Missing, *n* (%)	4 (2)	4 (3)		3 (2)	
Social support for health (HLQ 4)					
Mean (SD)	3.24 (0.60)	3.38 (0.53)	−0.08 (−0.17 to 0.02)	3.24 (0.61)	−0.04(− 0.14 to 0.07)
Missing, *n* (%)	5 (2)	4 (3)		5 (3)	
Ability to actively engage with healthcare providers (HLQ 6)					
Mean (SD)	3.84 (0.82)	3.87 (0.89)	−0.05 (−0.21 to 0.10)	3.85 (0.82)	−0.04 (− 0.20 to 0.14)
Missing, *n* (%)	6 (3)	4 (3)		6 (3)	
Understanding health information well enough to know what to do (HLQ 9)					
Mean (SD)	4.03 (0.77)	3.97 (0.85)	0.009 (−0.13 to 0.15)	4.02 (0.78)	0.04 (−0.12 to 0.20)
Missing, *n* (%)	6 (3)	4 (3)		6 (3)	
Self-efficacy (GSE)					
Mean (SD)	29.78 (5.69)	29.73 (6.14)	−0.22 (−1.22 to 0.78)	29.81 (5.75)	−0.02 (−1.16 to 1.13)
Missing, *n* (%)	7 (3)	4 (3)		7 (4)	
General health					
Mean (SD)	2.63 (0.93)	2.60 (0.82)	0.05 (−0.10 to 0.19)	2.61 (0.94)	0.06 (−0.11 to 0.22)
Missing, *n* (%)	1 (0.05)	1 (0.07)		1 (0.05)	
No. of seizure last year					
Mean (SD)	2.50 (11.89)	3.20 (10.21)	−0.72 (−3.20 to 1.75)	2.52 (12.04)	−0.63 (−3.50 to 2.24)
Missing, *n* (%)	36 (18)	28 (19)		33 (17)	
Side effects					
Mean (SD)	1.54 (0.76)	1.56 (0.83)	−0.03 (−0.18 to 0.11)	1.53 (0.77)	0.005 (−0–15 to 0.17)
Missing, *n* (%)	6 (3)	1 (0.07)		6 (3)	
Patient activation ^c^					
Mean (SD)	3.42 (0.65)	3.34 (0.77)	0.04 (−0.10 to 0.17)	3.42 (0.65)	0.001 (−0.15 to 0.15)
Missing, *n* (%)	6 (3)	4 (3)		6 (3)	
Patient activation ^d^					
Mean (SD)	3.22 (0.72)	3.12 (0.75)	0.01 (−0.13 to 0.16)	3.22 (0.73)	0.02 (−0.14 to 0.17)
Missing, *n* (%)	5 (2)	4 (3)		5 (2)	
Confidence					
Mean (SD)	1.39 (0.65)	1.33 (0.53)	0.03 (−0.9 to 0.16)	1.39 (0.65)	0.06 (−0.07 to 0.20)
Missing, *n* (%)	21 (10)	9 (6)		20 (10)	
Safety					
Mean (SD)	1.41 (0.70)	1.35 (0.56)	0.02 (−0.12 to 0.16)	1.41 (0.70)	0.07 (−0.09 to 0.23)
Missing, *n* (%)	37 (18)	14 (9)		36 (18)	
Satisfaction					
Mean (SD)	1.63 (0.68)	1.61 (0.59)	0.01 (−0.13 to 0.15)	1.63 (0.69)	0.05 (−0.11 to 0.20)
Missing, *n* (%)	35 (17)	18 (12)		34 (17)	

*SD* Standard deviation, *CI* Confidence interval, *WHO-5* WHO-5 well-being index, *HLQ* Health literacy questionnaire, *GSE* General self-efficacy scale

^a^The estimated intention-to-treat differences and 95% CIs were obtained after multiple linear regression adjusted for baseline measure

^b^The estimated per-protocol differences and 95% CIs were obtained after multiple linear regression adjusted for baseline measure, gender, age, education, cohabitation status, epilepsy duration, and seizures last year

^c^I am confident that I can tell when I need to get outpatient care

^d^I am confident I can figure out solutions when new situations or problems arise with my health condition

### Per-protocol and stratified analyses

Results from per-protocol analyses are shown in Tables 2 and 3. No statistically significant differences were found in either primary or secondary outcomes. Explorative stratified ITT analyses with stratification on gender and high/low health literacy did not change the results noticeably (Additional file 3, pages 1 to 9). After stratification on age in the low age group (median age below 45.7 years), the participants in the open access arm had fewer telephone consultations and emergency room visits, − 0.67 (95% CI: − 1.29 to − 0.04) and − 0.21 (95% CI: − 0.38 to − 0.03), respectively, compared to the standard arm. However, the mental well-being was lower in the open access arm than in the standard arm, difference: − 5.95 (95% CI: − 10.81 to − 1.08). No statistically significant differences were found in the high age group.

### Attrition and sensitivity analyses

Web (*N* = 648) and paper responders (*N* = 395) of the baseline research questionnaire were compared (Fig. 1). Web-responders were younger, had a higher level of education, and higher scores of health literacy and self-efficacy. No differences were found in gender, well-being, and general health. Responders (*N* = 352) to the follow-up research questionnaire were compared to non-responders (*N* = 241) by using data gathered at baseline. Participants who did not respond were younger, mean (SD) age 43.2 (17.0) years versus 49.0 (17.0) years, *P* = 0.0001. Furthermore, non-responders had a lower WHO-5 well-being score, mean (SD) 65.6 (19.1) versus 70.5 (18.8), *P* = 0.003, lower scores of the HLQ 6 “Ability to actively engage with healthcare providers”, mean (SD) 3.75 (0.90) versus 3.91 (0.83), *P* = 0.04, lower self-reported general health, *P* = 0.04, and lower level of education, *P* = 0.02. No differences were found with respect to gender, cohabitation status, “social support for health” (HLQ 4), “understand health information well enough to know what to do” (HLQ 9), self-efficacy (GSE), side effects, seizures during last year, and duration of epilepsy.

The sensitivity analyses showed that the difference in the WHO-5 score changed to not statistically significant if the missing data at follow-up were based on a 5-point lower baseline value in the standard arm, but were unchanged in the open access arm, 0.11 (95% CI: − 1.84 to 2.07) and a 5-point higher baseline value in the open access arm, but were unchanged in the standard arm, 0.18 (95% CI: − 1.81 to 2.17) (Additional file 3, page 10). The difference became stronger if the missing data was based on a 5-point higher baseline value in the standard arm, but were unchanged in the open access arm, − 3.83 (95% CI: − 5.78 to − 1.88) and a 5-point lower baseline value in the open access arm, but were unchanged in the standard arm, − 4.02 (95% CI: − 5.94 to − 2.10).

### Process evaluation in the open access arm

**Fig. 2:**
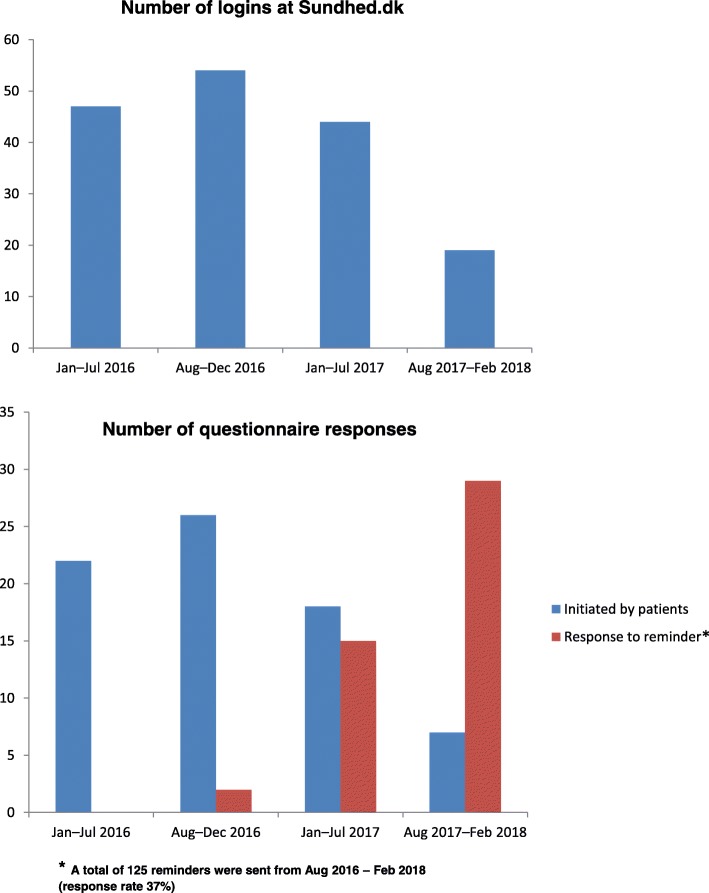
Activity in the open access arm (*N* = 346) during follow-up in terms of logins to the “My Epilepsy” web site at Sundhed.dk, questionnaires responses initiated by patients, and questionnaire responses of reminders sent to patients

Overall, activity in terms of number of logins to the “My Epilepsy” web site and questionnaires filled in initiated by the patients decreased during the follow-up period (Fig. 2). At the same time, an increased number of reminders were sent to patients; the response rate (37%) was, however, low.

## Discussion

The open access telePRO intervention showed no statistically significant differences in use of health care resources in terms of telephone consultations, outpatient visits, or hospital admissions compared to standard telePRO follow-up. The open access arm had a statistically significant, slightly lower mean number of emergency room visits. No statistically significant differences were found in clinical outcome measures such as mortality, number of seizures, and side effects. Further, for patient-centered outcome measures, the mental well-being was statistically significantly lower in the open access arm than in the standard arm, but there were no differences in general health status. No statistically significant differences were found in patient self-management and health service evaluation.

Effectiveness in terms of use of health care resources in an open access or a telemedicine intervention has been investigated in other studies, which have reported a lower number of outpatient visits in the intervention group than in the control group in patients with inflammatory bowel disease [37, 40, 41] and rheumatoid arthritis [42, 43]. However, in these studies the control group was offered pre-scheduled follow-up appointments in the clinic. This was not the case in our study, as the standard arm was another model of PRO-based follow-up, not traditional follow-up with fixed appointments. As a consequence, the contrast between the two arms was relatively low in our study, which may explain why there was small or no differences between the two types of PRO-based follow-up. Preferably, both study arms in this study should be compared with patients who received face-to-face follow-up. However, since standard telePRO was standard care for 2500 outpatients with epilepsy at Aarhus University Hospital in 2016, this option was limited. By including patients already attending standard telePRO follow-up, we took advantage of recruiting a large proportion of prevalent patients within 6 months. Although, another study could be based on new patients, as they could be randomized to the two different PRO-based follow-up arms used in this study or traditional face-to-face follow-up. However, this design was not customized according to clinical practice in the department and recruitment of new patients would require a much longer recruitment period, as the department receives approximately only 250 new patients yearly. Still, we found it relevant to investigate whether a more tailored individual follow-up method initiated by the patients could lead to further benefits for the patients and the health care system, as the fixed-interval PRO-based model is primary driven by clinicians who determine the questionnaire intervals. Our findings related to clinical outcomes are in accordance with results from other studies in patients with rheumatoid arthritis [42, 43], inflammatory bowel disease [41], and chronic obstructive pulmonary disease [44], which also found no differences in disease activity. In addition, a review reported similar health-related quality of life and psychological outcomes in patient-initiated follow-up compared to traditional follow-up [18]. Patients in the open access arm in our study reported a statistically lower self-reported mental well-being than those in the standard arm; however, the difference was small and probably not clinically significant, since a clinically relevant change on the WHO-5 scale is considered to be 10 points [27]. Further, the measurement error of WHO-5 has been estimated to be around 20 points in an epilepsy outpatient population, and this should be taken into consideration when using the scale to measure change over time [45].

The main strength of this study was the pragmatic randomized design customized according to real life implementation of PRO-based follow-up in clinical practice. Furthermore, the study included a large study sample, and loss to follow-up in the analyses of the primary outcome was limited. However, some limitations should be noted. The baseline level of the HLQ subscales and the GSE in the epilepsy population was nearly the same as the in Danish population as a whole [31, 32]; thus a ceiling effect occurred, and it became difficult to observe improvement in these constructs over time. Another limitation was the low response rate of the questionnaires at 18-month follow-up. Only approximately 60% of the patients in both arms responded; thus, selection bias cannot be ruled out. The sensitivity analyses of self-reported WHO-5 well-being showed that the results could potentially be both underestimated or moved toward the null hypothesis of no effect. All eligible patients were referred to standard telePRO; therefore, we decided to use a pre-randomization design with few inclusion criteria. However, this study only included patients who were able to fill in the questionnaire via the Internet. As shown in Fig. 1, this is a selected standard telePRO group of patients because 395 paper responders (38%) were excluded. This should be taken into consideration in the generalization of the results.

Non-adherence to the use of health technology interventions is a common problem [46]. Participants make use of the intervention differently and not all continue to use the intervention as intended [46]. Data from Fig. 2 indicate that the number of patients who actually used the open access website was low. It is important to explain program failures if the intervention does not function as intended. Greenhalgh et al. describe the complexities of predicting if or how people engage with health technology in a new theoretical framework [47]. The framework includes seven domains: the condition, the technology, the value proposition, the adopter system, the organization, the wider societal context, and the interaction between these domains over time [47]. We have used elements of this framework to discuss challenges related to the open access intervention. At the organizational level, much effort was put into developing the intervention, but the implementation strategy was probably insufficient, leading to issues related to knowledge and confidence in using the intervention as intended by the patients. Information about the intervention was mailed to the participants only once during the follow-up period, and the participants were expected to take action by themselves if they declined to participate in the open access arm. At the individual patient level, the open access intervention demanded some self-management skills because the patients were expected to actively interact with the health care system. However, patients are only activated if “they understand their role in the health care process and have the knowledge, skills and confidence to carry it out” [2]. There could be resistance by the patients regarding filling in a questionnaire in order to get in contact with the clinic, as they might have found it easier to call the clinic if they needed to talk to a clinician. Resistance could also be related to technical issues, for example, the login procedure to the open access website required some extra steps, and technical problems were experienced by some patients. Qualitative data regarding the patient perspective will be further investigated.

## Conclusion

There is growing need for health care strategies to manage more effective and patient-centered care. This study did not find, as hypothesized, less use of health care resources or improved patient self-management or satisfaction in the patient-initiated PRO-based initiative compared to fixed-interval PRO-based follow-up. Patient-initiated PRO-based follow-up may be used as an alternative to fixed-interval PRO-based follow-up in epilepsy outpatients who prefer this approach, but there is insufficient evidence for recommending a system-wide shift to patient-initiated PRO-based follow-up. How patients are allocated to this health care service is important, and individuals’ self-management skills should be taken into consideration. Further work should explore the effects of using a patient-initiated PRO-based intervention in clinical practice, preferably, comparing these patients with patients using a fixed-appointment follow-up procedure.

## Additional files

Additional file 1: Information for Reporting Randomized Controlled Trials With Patient report Outcomes. (PDF 1129 kb)

Additional file 2: Disease-specific epilepsy questionnaire. (PDF 1501 kb)

Additional file 3: Stratified analyses, sensitivity analyses, and the randomization computer code. (PDF 624 kb)

## Data Availability

Access to the protocol for this study can be found as reference [22] and via this link: 10.1186/s12913-017-2015-8. Please contact the corresponding author for further guidance regarding data request.

## Abbreviations

CI: Confidence interval
CONSORT: Consolidated Standard of Reporting Trial
GH: General health
GSE: General Self-Efficacy Scale
HLQ: Health Literacy Questionnaire
IQR: Interquartile range
ITT: Intention-to-treat
PAM: Patient Activation Measure
PRO: Patient-reported outcome
SD: Standard deviation
WHO-5: WHO-5 Well-Being Index
