# Supplementary material for: Patient-initiated versus fixed-interval patient-reported outcome-based follow-up in outpatients with epilepsy: a pragmatic randomized controlled trial
Source: J Patient Rep Outcomes. 2019 Sep 13;3:61. doi: 10.1186/s41687-019-0151-0 (PMC6744536; doi:10.1186/s41687-019-0151-0)
Supplement: Supplementary file 1 — Information for Reporting Randomized Controlled Trials With Patient report Outcomes. (PDF 1129 kb) [file 41687_2019_151_MOESM1_ESM.pdf]

Table 1. Information for Reporting Randomized Controlled Trials With Patient reported Outcomes

Section/Topic	Item	CONSORT 2010 Statement Checklist Item	PRO-Specific Extensions Are Prefaced by the letter *P*	
		**Title and Abstract**		
	1a	Identification as a randomized trial in the title		Page 1
	1b	Structured summary of trial design, methods, results, and conclusions (for specific guidance see CONSORT for abstracts)[3]	P1b: The PRO should be identified in the abstract as a primary or secondary outcome	Page 1-2
		**Introduction**		
Background and objectives	2a	Scientific background and explanation of rationale	Including background and rationale for PRO assessment	Page 3-4
	2b	Specific objectives or hypotheses	P2b: The PRO hypothesis should be stated and relevant domains identified, if applicable	Page 4-5
		**Methods**		
Trial design	3a	Description of trial design (such as parallel, factorial), including allocation ratio		Page 5
	3b	Important changes to methods after trial commencement (such as eligibility criteria), with reasons		N/A
Participants	4a	Eligibility criteria for participants	Not PRO-specific, unless the PROs were used in eligibility or stratification criteria	Page 5
	4b	Settings and locations where the data were collected		Page 6
Interventions	5	The interventions for each group with sufficient details to allow replication, including how and when they were actually administered		Page 6-8
Outcomes	6a	Completely defined prespecified primary and secondary outcome measures, including how and when they were assessed	P6a: Evidence of PRO instrument validity and reliability should be provided or cited if available including the person completing the PRO and methods of data collection (paper, telephone, electronic, other)	Page 8-9
	6b	Any changes to trial outcomes after the trial commenced, with reasons		N/A
Sample size	7a	How sample size was determined	Not required for PRO unless it is a primary study outcome	Page 10
	7b	When applicable, explanation of any interim analyses and stopping guidelines		N/A
		**Randomization**		
Sequence generation	8a	Method used to generate the random allocation sequence		Page 6
	8b	Type of randomization; details of any restriction (such as blocking and block size)		Page 6
Allocation concealment mechanism	9	Mechanism used to implement the random allocation sequence (such as sequentially numbered containers), describing any steps taken to conceal the sequence until interventions were assigned		Page 5-6
Implementation	10	Who generated the random allocation sequence, who enrolled participants, and who assigned participants to interventions		Page 6
Blinding	11a	If done, who was blinded after assignment to interventions (for example, participants, care providers, those assessing outcomes) and how		Page 6
	11b	If relevant, description of the similarity of interventions		N/A
Statistical methods	12a	Statistical methods used to compare groups for primary and secondary outcomes	P12a: Statistical approaches for dealing with missing data are explicitly stated	Page 10-11
	12b	Methods for additional analyses, such as subgroup analyses and adjusted analyses		Page 11
		**Results**		
Participant flow (a diagram is strongly recommended)	13a	For each group, the numbers of participants who were randomly assigned, received intended treatment, and were analyzed for the primary outcome	The number of PRO outcome data at baseline and at subsequent time points should be made transparent	Figure 1
	13b	For each group, losses and exclusions after randomization, together with reasons		Figure 1
Recruitment	14a	Dates defining the periods of recruitment and follow-up		Page 12
	14b	Why the trial ended or was stopped		N/A
Baseline data	15	A table showing baseline demographic and clinical characteristics for each group	Including baseline PRO data when collected	Table 1
Numbers analyzed	16	For each group, number of participants (denominator) included in each analysis and whether the analysis was by original assigned groups	Required for PRO results	Figure 1 + Table 2 and 3
Outcomes and estimation	17a	For each primary and secondary outcome, results for each group, the estimated effect size, and its precision (such as 95% confidence interval)	For multidimensional PRO results from each domain and time point	Page 12-13 + Table 2 and 3
	17b	For binary outcomes, presentation of both absolute and relative effect sizes is recommended		N/A
Ancillary analyses	18	Results of any other analyses performed, including subgroup analyses and adjusted analyses, distinguishing prespecified from exploratory	Including PRO analyses, where relevant	Page 13-14
Harms	19	All important harms or unintended effects in each group (for specific guidance see CONSORT for harms)		N/A
		**Discussion**		
Limitations	20	Trial limitations, addressing sources of potential bias, imprecision, and, if relevant, multiplicity of analyses	P20/21: PRO–specific limitations and implications for generalizability and clinical practice	Page 15-17
Generalizability	21	Generalizability (external validity, applicability) of the trial findings		Page 16
Interpretation	22	Interpretation consistent with results, balancing benefits and harms, and considering other relevant evidence	PRO data should be interpreted in relation to clinical outcomes including survival data, where relevant	Page 15-17
		**Other Information**		
Registration	23	Registration number and name of trial registry		In Abstract
Protocol	24	Where the full trial protocol can be accessed, if available		Page 18
Funding	25	Sources of funding and other support (such as supply of drugs), role of funders		Page 18

Reproduced from *Reporting of Patient-Reported outcomes in Randomized Trials: The CONSORT PRO Extension. JAMA, February 27, 2013_Vol309(8)*

Copyright © 2013 American Medical association. All rights reserved."
