# Supplementary material for: Patient-initiated versus fixed-interval patient-reported outcome-based follow-up in outpatients with epilepsy: a pragmatic randomized controlled trial
Source: J Patient Rep Outcomes. 2019 Sep 13;3:61. doi: 10.1186/s41687-019-0151-0 (PMC6744536; doi:10.1186/s41687-019-0151-0)
Supplement: Supplementary file 3 — Stratified analyses, sensitivity analyses, and the randomization computer code. (PDF 624 kb) [file 41687_2019_151_MOESM3_ESM.pdf]

Additional file 3

Supplemental analyses

Analyses stratified on age

AGE HIGH (Age at baseline ≥ 45.7 years)

Health care utilization during an 18-month follow-up period among outpatients with epilepsy

**Primary outcomes**	**Open access (ITT)** *N* **= 169**	**Control** *N* **= 124**	**ITT Mean difference (95%CI)**
Outpatient visits [a]			
Mean (SD)	0.41 (0.97)	0.38 (0.88)	0.04 (−0.18 to 0.25)
Median (Range)	0 (0–7)	0 (0–6)	
Telephone consultations [a]			
Mean (SD)	0.86 (1.62)	0.85 (1.39)	0.01 (−0.33 to 0.35)
Median (Range)	0 (0–10)	1 (0–10)	
Hospitalizations [a]			
Mean (SD)	0.08 (0.36)	0.06 (0.26)	0.02 (−0.05 to 0.09)
Median (Range)	0 (0–3)	0 (0–2)	
Emergency room visits [b]			
Mean (SD)	0.08 (0.41)	0.10 (0.42)	−0.02 (−0.12 to 0.08)
Median (Range)	0 (0–4)	0 (0–3)	

[a] at the Department of Neurology, Aarhus University Hospital, [b] at Aarhus University Hospital
SD: Standard deviation; CI: Confidence interval; ITT: Intention-to-treat
The estimated IIT mean differences and 95% CIs were obtained after simple linear regression by using the bootstrap method with 1000 replications.

AGE LOW (Age at baseline < 45.7 years)

Health care utilization during an 18-month follow-up period among outpatients with epilepsy

**Primary outcomes**	**Open access (ITT)** *N* **= 174**	**Control** *N* **= 119**	**ITT Mean difference (95%CI)**
Outpatient visits [a]			
Mean (SD)	0.48 (0.93)	0.45 (0.84)	0.03 (−0.18 to 0.24)
Median (Range)	0 (0–6)	0 (0–5)	
Telephone consultations [a]			
Mean (SD)	1.11 (2.09)	1.78 (3.16)	−0.67 (−1.29 to −0.04)
Median (Range)	0 (0–12)	1 (0–22)	
Hospitalizations [a]			
Mean (SD)	0.02 (0.18)	0.12 (0.65)	−0.09 (−0.22 to 0.03)
Median (Range)	0 (0–2)	0 (0–5)	
Emergency room visits [b]			
Mean (SD)	0.06 (0.34)	0.27 (0.94)	−0.21 (−0.38 to −0.03)
Median (Range)	0 (0–3)	0 (0–7)	

[a] at the Department of Neurology, Aarhus University Hospital, [b] at Aarhus University Hospital
SD: Standard deviation; CI: Confidence interval; ITT: Intention-to-treat
The estimated IIT mean differences and 95% CIs were obtained after simple linear regression by using the bootstrap method with 1000 replications.

Additional file 3

Analyses stratified on age

AGE HIGH (Age at baseline ≥ 45.7 years)

Patient-reported outcomes measured 18 months after randomization among outpatients with epilepsy

Secondary outcomes	*N*	Open access (ITT)	*N*	Control	ITT: Difference [a] at 18-mo. follow-up (95%CI)
Well-being (WHO-5)<br>Mean (SD)	107	69.83(19.46)	89	69.48(17.69)	−1.36 (−5.59 to 2.87)
Self-efficacy (GSE)<br>Mean (SD)	105	30.00(5.52)	90	29.56(5.95)	−0.03 (−1.33 to 1.27)
HLQ 4<br>Mean (SD)	106	3.24(0.61)	90	3.35(0.53)	−0.04 (−0.17 to 0.09)
HLQ 6<br>Mean (SD)	105	3.89(0.79)	91	3.85(0.90)	−0.07 (−0.27 to 0.14)
HLQ 9<br>Mean (SD)	105	4.01(0.79)	90	3.90(0.88)	−0.007 (−0.19 to 0.17)
General health<br>Mean (SD)	109	2.68(0.88)	92	2.66(0.73)	0.04 (−0.14 to 0.23)
No. of seizure last year<br>Mean (SD)	93	2.71(13.00)	76	3.58(11.00)	−0.69 (−4.02 to 2.67)
Treatment side effects<br>Mean (SD)	108	1.53(0.77)	92	1.55(0.89)	−0.05(−0.26 to 0.15)
Patient activation [b]<br>Mean (SD)	108	3.39(0.61)	91	3.27(0.79)	0.08 (−0.09 to 0.25)
Patient activation [c]<br>Mean (SD)	108	3.23(0.68)	91	3.10(0.80)	0.03(−0.15 to 0.22)
Confidence<br>Mean (SD)	97	1.38(0.64)	87	1.30(0.51)	0.01(−0.14 to 0.17)
Safety<br>Mean (SD)	88	1.43(0.67)	82	1.28(0.48)	0.10(−0.07 to 0.27)
Satisfaction<br>Mean (SD)	91	1.65(0.69)	79	1.59(0.59)	0.05 (−0.14 to 0.24)

[a] The estimated intention-to-treat differences and 95% CIs were obtained after multiple linear regression adjusted for baseline measure
[b] I am confident that I can tell when I need to get outpatient care
[c] I am confident I can figure out solutions when new situations or problems arise with my health condition
SD: Standard deviation; CI: Confidence interval; ITT: Intention-to-treat; WHO-5: WHO-5 Well-being Index; GSE: General Self-efficacy Scale; HLQ: Health Literacy Questionnaire

Additional file 3

Analyses stratified on age

AGE LOW (Age at baseline < 45.7 years)

Patient-reported outcomes measured 18 months after randomization among outpatients with epilepsy

**Secondary outcomes**	***N***	**Open access (ITT)**	***N***	**Control**	**ITT: Difference [a] at 18-mo. follow-up (95%CI)**
Well-being (WHO-5)<br>Mean (SD)	91	63.65(19.01)	57	68.98(18.66)	−5.95 (−10.81 to −1.08)
Self-efficacy (GSE)<br>Mean (SD)	90	29.52(5.90)	56	30.02(6.47)	−0.50 (−2.11 to 1.12)
HLQ 4<br>Mean (SD)	88	3.25(0.61)	53	3.42(0.54)	−0.14 (−0.29 to 0.006)
HLQ 6<br>Mean (SD)	88	3.80(0.82)	55	3.90(0.87)	−0.02 (−0.26 to 0.21)
HLQ 9<br>Mean (SD)	88	4.04(0.76)	55	4.07(0.82)	0.005 (−0.22 to 0.23)
General health<br>Mean (SD)	92	2.57(0.99)	57	2.51(0.95)	0.05 (−0.19 to 0.30)
No. of seizure last year<br>Mean (SD)	73	2.23(10.39)	46	2.52(8.83)	−0.58 (−4.27 to 3.12)
Treatment side effects<br>Mean (SD)	88	1.55(0.76)	57	1.56(0.71)	−0.002(−0.20 to 0.19)
Patient activation [b]<br>Mean (SD)	88	3.45(0.69)	55	3.45(0.74)	−0.06 (−0.29 to 0.17)
Patient activation [c]<br>Mean (SD)	89	3.20(0.77)	55	3.16 (0.66)	−0.03(−0.27 to 0.20)
Confidence<br>Mean (SD)	84	1.40(0.66)	54	1.37(0.56)	0.05(−0.15 to 0.25)
Safety<br>Mean (SD)	77	1.39(0.73)	54	1.46(0.66)	−0.09(−0.33 to 0.15)
Satisfaction<br>Mean (SD)	76	1.61(0.67)	53	1.62(0.60)	−0.03 (−0.24 to 0.18)

[a] The estimated intention-to-treat differences and 95% CIs were obtained after multiple linear regression adjusted for baseline measure
[b] I am confident that I can tell when I need to get outpatient care
[c] I am confident I can figure out solutions when new situations or problems arise with my health condition
SD: Standard deviation; CI: Confidence interval; ITT: Intention-to-treat; WHO-5: WHO-5 Well-being Index; GSE: General Self-efficacy Scale; HLQ: Health Literacy Questionnaire

Additional file 3

Analyses stratified on gender

FEMALE

Health care utilization during an 18-month follow-up period among outpatients with epilepsy

**Primary outcomes**	**Open access (ITT)** ***N* = 162**	**Control** ***N* = 130**	**ITT Mean difference (95%CI)**
Outpatient visits [a]			
Mean (SD)	0.55 (1.01)	0.52 (1.04)	0.03 (−0.20 to 0.26)
Median (Range)	0 (0–7)	0 (0–6)	
Telephone consultations [a]			
Mean (SD)	1.27 (2.17)	1.62 (2.93)	−0.36 (−0.95 to 0.24)
Median (Range)	0 (0–12)	1 (0–22)	
Hospitalizations [a]			
Mean (SD)	0.02 (0.19)	0.11 (0.56)	−0.08 (−0.18 to 0.01)
Median (Range)	0 (0–2)	0 (0–5)	
Emergency room visits [b]			
Mean (SD)	0.04 (0.22)	0.19 (0.75)	−0.16 (−0.29 to −0.02)
Median (Range)	0 (0–2)	0 (0–7)	

[a] at the Department of Neurology, Aarhus University Hospital, [b] at Aarhus University Hospital
SD: Standard deviation; CI: Confidence interval; ITT: Intention-to-treat
The estimated IIT mean differences and 95% CIs were obtained after simple linear regression by using the bootstrap method with 1000 replications.

MALE

Health care utilization during an 18-month follow-up period among outpatients with epilepsy

**Primary outcomes**	**Open access (ITT)** ***N* = 181**	**Control** ***N* = 113**	**ITT Mean difference (95%CI)**
Outpatient visits [a]			
Mean (SD)	0.36 (0.88)	0.29 (0.58)	0.07 (−0.10 to 0.23)
Median (Range)	0 (0–6)	0 (0–2)	
Telephone consultations [a]			
Mean (SD)	0.74 (1.54)	0.94 (1.71)	−0.20 (−0.59 to 0.19)
Median (Range)	0 (0–10)	1 (0–10)	
Hospitalizations [a]			
Mean (SD)	0.07 (0.35)	0.06 (0.41)	0.01 (−0.08 to 0.10)
Median (Range)	0 (0–3)	0 (0–4)	
Emergency room visits [b]			
Mean (SD)	0.10 (0.48)	0.18 (0.70)	−0.07 (−0.22 to 0.07)
Median (Range)	0 (0–4)	0 (0–6)	

[a] at the Department of Neurology, Aarhus University Hospital, [b] at Aarhus University Hospital
SD: Standard deviation; CI: Confidence interval; ITT: Intention-to-treat
The estimated IIT mean differences and 95% CIs were obtained after simple linear regression by using the bootstrap method with 1000 replications.

Additional file 3

Analyses stratified on gender

FEMALE

Patient-reported outcomes measured 18 months after randomization among outpatients with epilepsy

**Secondary outcomes**	***N***	**Open access (ITT)**	***N***	**Control**	**ITT: Difference [a] at 18-mo. follow-up (95%CI)**
Well-being (WHO-5)<br>Mean (SD)	91	66.24(18.96)	79	67.24(18.91)	−2.94 (−7.32 to 1.44)
Self-efficacy (GSE)<br>Mean (SD)	87	29.29(5.86)	80	29.21(6.19)	−0.46 (−1.81 to 0.89)
HLQ 4<br>Mean (SD)	89	3.31(0.58)	78	3.35(0.59)	−0.009 (−0.14 to 0.12)
HLQ 6<br>Mean (SD)	87	3.89(0.77)	79	3.93(0.79)	−0.11 (−0.29 to 0.07)
HLQ 9<br>Mean (SD)	87	4.06(0.81)	79	4.03(0.76)	−0.005 (−0.18 to 0.17)
General health<br>Mean (SD)	93	2.59(0.89)	80	2.70(0.83)	−0.05 (−0.26 to 0.16)
No. of seizure last year<br>Mean (SD)	78	2.32(9.60)	65	3.60(10.80)	−1.51 (−4.97 to 1.95)
Treatment side effects<br>Mean (SD)	90	1.51(0.71)	80	1.59(0.85)	−0.14 (−0.32 to 0.05)
Patient activation [b]<br>Mean (SD)	90	3.46(0.66)	79	3.39(0.72)	0.08 (−0.12 to 0.27)
Patient activation [c]<br>Mean (SD)	91	3.26(0.73)	79	3.08(0.73)	0.12 (−0.08 to 0.32)
Confidence<br>Mean (SD)	86	1.34(0.59)	76	1.29(0.51)	−0.02(−0.18 to 0.13)
Safety<br>Mean (SD)	80	1.34(0.57)	74	1.36(0.56)	−0.08(−0.25 to 0.10)
Satisfaction<br>Mean (SD)	82	1.65(0.64)	69	1.58(0.55)	0.04 (−0.15 to 0.24)

[a] The estimated intention-to-treat differences and 95% CIs were obtained after multiple linear regression adjusted for baseline measure

[b] I am confident that I can tell when I need to get outpatient care

[c] I am confident I can figure out solutions when new situations or problems arise with my health condition

SD: Standard deviation; CI: Confidence interval; ITT: Intention-to-treat; WHO-5: WHO-5 Well-being Index; GSE: General Self-efficacy Scale; HLQ: Health Literacy Questionnaire

Additional file 3

Analyses stratified on gender

MALE

Patient-reported outcomes measured 18 months after randomization among outpatients with epilepsy

**Secondary outcomes**	***N***	**Open access (ITT)**	***N***	**Control**	**ITT: Difference [a] at 18-mo. follow-up (95%CI)**
Well-being (WHO-5)<br>Mean (SD)	107	67.63(19.93)	67	71.70(16.70)	−3.74 (−8.42 to 0.93)
Self-efficacy (GSE)<br>Mean (SD)	90	30.18(5.55)	66	30.36(6.06)	−0.09 (−1.59 to 1.42)
HLQ 4<br>Mean (SD)	105	3.19(0.62)	65	3.41(0.50)	−0.15 (−0.29 to −0.002)
HLQ 6<br>Mean (SD)	106	3.82(0.83)	67	3.79(0.99)	0.05 (−0.19 to 0.30)
HLQ 9<br>Mean (SD)	106	3.99(0.75)	66	3.89(0.96)	0.04 (−0.18 to 0.27)
General health<br>Mean (SD)	108	2.66(0.97)	69	2.49(0.80)	0.12 (−0.09 to 0.34)
No. of seizure last year<br>Mean (SD)	88	2.66(13.66)	57	2.70(9.57)	0.24 (−2.27 to 2.75)
Treatment side effects<br>Mean (SD)	106	1.56(0.81)	69	1.52(0.80)	0.05 (−0.17 to 0.28)
Patient activation [b]<br>Mean (SD)	106	3.39(0.64)	67	3.28(0.83)	−0.007 (−0.21 to 0.19)
Patient activation [c]<br>Mean (SD)	106	3.18(0.74)	67	3.18 (0.78)	−0.10(−0.32 to 0.11)
Confidence<br>Mean (SD)	95	1.44(0.70)	65	1.37(0.55)	0.08 (−0.12 to 0.27)
Safety<br>Mean (SD)	85	1.48(0.80)	62	1.34(0.57)	0.12(−0.10 to 0.34)
Satisfaction<br>Mean (SD)	85	1.61(0.73)	63	1.63(0.63)	−0.02 (−0.23 to 0.18)

[a] The estimated intention-to-treat differences and 95% CIs were obtained after multiple linear regression adjusted for baseline measure
[b] I am confident that I can tell when I need to get outpatient care
[c] I am confident I can figure out solutions when new situations or problems arise with my health condition
SD: Standard deviation; CI: Confidence interval; ITT: Intention-to-treat; WHO-5: WHO-5 Well-being Index; GSE: General Self-efficacy Scale; HLQ: Health Literacy Questionnaire

Additional file 3

Analyses stratified on health literacy 'Social support for health" (HLQ4)

HLQ 4 HIGH (HLQ 4 scale measured at baseline ≥ 3.4)

Health care utilization during an 18-month follow-up period among outpatients with epilepsy

**Primary outcomes**	**Open access (ITT)** ***N*** **= 183**	**Control** ***N*** **= 136**	**ITT Mean difference (95%CI)**
Outpatient visits [a]			
Mean (SD)	0.43 (0.91)	0.40 (0.93)	0.03 (−0.16 to 0.23)
Median (Range)	0 (0–6)	0 (0–6)	
Telephone consultations [a]			
Mean (SD)	1.04 (1.78)	1.31 (2.18)	−0.27 (−0.72 to 0.19)
Median (Range)	0 (0–10)	1 (0–12)	
Hospitalizations [a]			
Mean (SD)	0.03 (0.21)	0.05 (0.49)	−0.02 (−0.07 to 0.03)
Median (Range)	0 (0–2)	0 (0–2)	
Emergency room visits [b]			
Mean (SD)	0.05 (0.24)	0.15 (0.45)	−0.11 (−0.19 to −0.02)
Median (Range)	0 (0 – 2)	0 (0 – 3)	

[a] at the Department of Neurology, Aarhus University Hospital, [b] at Aarhus University Hospital
SD: Standard deviation; CI: Confidence interval; ITT: Intention-to-treat
The estimated IIT mean differences and 95% CIs were obtained after simple linear regression by using the bootstrap method with 1000 replications.

HLQ 4 LOW (HLQ 4 scale measured at baseline < 3.4)

Health care utilization during an 18-month follow-up period among outpatients with epilepsy

**Primary outcomes**	**Open access (ITT)** ***N*** **= 175**	**Control** ***N*** **= 113**	**ITT Mean difference (95%CI)**
Outpatient visits [a]			
Mean (SD)	0.45 (0.96)	0.42 (0.75)	0.03 (−0.17 to 0.23)
Median (Range)	0 (0–7)	0 (0–3)	
Telephone consultations [a]			
Mean (SD)	0.92 (1.92)	1.32 (2.76)	−0.40 (−0.98 to 0.18)
Median (Range)	0 (0–12)	1 (0–22)	
Hospitalizations [a]			
Mean (SD)	0.06 (0.34)	0.12 (0.67)	−0.06 (−0.19 to0.07)
Median (Range)	0 (0–3)	0 (0–5)	
Emergency room visits [b]			
Mean (SD)	0.10 (0.48)	0.22 (0.94)	−0.12 (−0.31 to 0.06)
Median (Range)	0 (0–4)	0 (0–7)	

[a] at the Department of Neurology, Aarhus University Hospital, [b] at Aarhus University Hospital
SD: Standard deviation; CI: Confidence interval; ITT: Intention-to-treat
The estimated IIT mean differences and 95% CIs were obtained after simple linear regression by using the bootstrap method with 1000 replications.

Additional file 3

Analyses stratified on health literacy 'Social support for health" (HLQ4)

HLQ 4 HIGH (HLQ 4 scale measured at baseline ≥ 3.4)

Patient-reported outcomes measured 18 months after randomization among outpatients with epilepsy

Secondary outcomes	N	Open access (ITT)	N	Control	ITT: Difference [a] at 18-mo. follow-up (95%CI)
Well-being (WHO-5) Mean (SD)	115	70.64(17.39)	89	69.29(18.01)	−3.68 (−7.39 to 0.03)
Self-efficacy (GSE) Mean (SD)	112	31.01(5.72)	90	30.96(5.42)	−0.03 (−1.32 to 1.26)
General health Mean (SD)	115	2.42(0.87)	92	2.41(0.71)	0.06 (−0.12 to 0.25)
No. of seizure last year Mean (SD)	98	2.19(11.36)	78	2.69(9.84)	−0.34 (−3.60 to 2.92)
Treatment side effects Mean (SD)	115	1.46(0.72)	92	1.52(0.84)	−0.05(−0.23 to 0.13)
Patient activation [b] Mean (SD)	113	3.54(0.64)	90	3.46(0.75)	0.06 (−0.12 to 0.23)
Patient activation [c] Mean (SD)	113	3.33(0.70)	90	3.21(0.79)	−0.003(−0.20 to 0.19)
Confidence Mean (SD)	104	1.31(0.58)	86	1.29(0.51)	−0.03(−0.17 to 0.11)
Safety Mean (SD)	98	1.34(0.67)	82	1.30(0.51)	0.02(−0.16 to 0.19)
Satisfaction Mean (SD)	98	1.51(0.63)	81	1.56(0.63)	−0.03 (−0.21 to 0.15)

[a] The estimated intention-to-treat differences and 95% CIs were obtained after multiple linear regression adjusted for baseline measure
[b] I am confident that I can tell when I need to get outpatient care
[c] I am confident I can figure out solutions when new situations or problems arise with my health condition
SD: Standard deviation; CI: Confidence interval; ITT: Intention-to-treat; WHO-5: WHO-5 Well-being Index; GSE: General Self-efficacy Scale; HLQ: Health Literacy Questionnaire

Additional file 3

Analyses stratified on health literacy 'Social support for health" (HLQ4)

HLQ 4 LOW (HLQ 4 scale measured at baseline < 3.4)

Patient-reported outcomes measured 18 months after randomization among outpatients with epilepsy

Secondary outcomes	N	Open access (ITT)	N	Control	ITT: Difference [a] at 18-mo. follow-up (95%CI)
Well-being (WHO-5) Mean (SD)	93	63.31(20.62)	61	64.98(20.63)	−2.73 (−8.08 to 2.62)
Self-efficacy (GSE) Mean (SD)	93	28.32(5.04)	59	27.76(6.60)	−0.26 (−1.83 to 1.31)
General health Mean (SD)	96	2.85(0.93)	61	2.87(0.92)	0.03 (−0.20 to 0.26)
No. of seizure last year Mean (SD)	81	2.60(11.99)	47	3.83(10.58)	−2.04 (−5.43 to 1.35)
Treatment side effects Mean (SD)	91	1.60(0.80)	61	1.61(0.78)	−0.02(−0.25 to 0.21)
Patient activation [b] Mean (SD)	93	3.28(0.61)	58	3.17(0.78)	0.06 (−0.16 to 0.29)
Patient activation [c] Mean (SD)	94	3.11(0.71)	58	3.00 (0.68)	0.03(−0.20 to 0.26)
Confidence Mean (SD)	85	1.51(0.72)	57	1.39(0.56)	0.10(−0.11 to 0.32)
Safety Mean (SD)	73	1.49(0.71)	56	1.43(0.63)	0.01(−0.22 to 0.25)
Satisfaction Mean (SD)	77	1.75(0.71)	53	1.68(0.51)	0.05 (−0.17 to 0.28)

[a] The estimated intention-to-treat differences and 95% CIs were obtained after multiple linear regression adjusted for baseline measure
[b] I am confident that I can tell when I need to get outpatient care
[c] I am confident I can figure out solutions when new situations or problems arise with my health condition
SD: Standard deviation; CI: Confidence interval; ITT: Intention-to-treat; WHO-5: WHO-5 Well-being Index; GSE: General Self-efficacy Scale; HLQ: Health Literacy Questionnaire

Additional file 3

Sensitivity analyses of WHO-5 Well-being Index

Sensitivity analyses were performed to establish the impact of missing self-reported data in WHO-5 Well-Being Index. The response rate of the follow-up questionnaire was approximately 60%.

Mean and standard deviation (SD) of the WHO-5 Well-being Index measured at baseline and at follow-up (18 months after randomization):

	**Intervention arm (open access telePRO)** ***N* = 346**	**Control arm (standard telePRO)** ***N* = 247**
Baseline WHO-5		
Mean (SD)	68.9 (18.9)	68.0 (19.4)
Missing, *n (%)*	10 (3)	3 (1)
Follow-up WHO-5		
Mean (SD)	67.0 (19.45)	69.3 (18.01)
Missing, *n (%)*	144 (42)	97 (39)

If the WHO-5 score was missing at follow-up, the score was imputed by using the WHO-5 score from the baseline questionnaire. Four scenarios regarding the imputed follow-up values were considered:

Analysis 1
Intervention arm: Baseline WHO reduced with 5 points
Control arm: WHO-5 baseline unchanged

Analysis 2
Intervention arm: WHO-5 baseline unchanged
Control arm: Baseline WHO reduced with 5 points

Analysis 3
Intervention arm: Baseline WHO increased with 5 points
Control arm: WHO-5 baseline unchanged

Analysis 4
Intervention arm: WHO-5 baseline unchanged
Control arm: Baseline WHO increased with 5 points

Then, between-arm differences in the WHO-5 score at follow-up were analyzed by multiple linear regression adjusted for the baseline WHO-5 value. Patients who died or emigrated (*N* = 7) or had missing WHO-5 scores at both baseline and follow-up (*N*=3) were not included in the analyses.

**WHO-5 Well-being**	**Intervention arm** ***N* = 341**	**Control arm** ***N* = 242**	**Difference at 18 mo. follow-up (95% Confidence interval)**
**Analysis 1**			
WHO-5 mean (SD)	64.4 (19.5)	67.7 (18.6)	−4.02 (−5.94 to −2.10)
**Analysis 2**			
WHO-5 mean (SD)	66.5 (19.3)	65.8 (19.0)	0.11 (−1.84 to 2.07)
**Analysis 3**			
WHO-5 mean (SD)	68.5 (19.3)	67.7 (18.6)	0.18 (−1.81 to 2.17)
**Analysis 4**			
WHO-5 mean (SD)	66.5 (19.3)	69.7 (18.5)	−3.83 (−5.78 to −1.88)

Additional file 3

The randomization computer code

The randomization was programmed in the computer language PHP:

```
$fraction= round(mt_rand() / mt_getrandmax(),4);  //generates random decimal fraction between 0.000 and 1.000

IF($fraction>0.45)

$allocation='OpenAccess';

ELSE

$allocation='Normal';
```
